# Independent and Combined Effects of Telomere Shortening and mtDNA^4977^ Deletion on Long-term Outcomes of Patients with Coronary Artery Disease

**DOI:** 10.3390/ijms20215508

**Published:** 2019-11-05

**Authors:** Cecilia Vecoli, Andrea Borghini, Silvia Pulignani, Antonella Mercuri, Stefano Turchi, Eugenio Picano, Maria Grazia Andreassi

**Affiliations:** CNR Institute of Clinical Physiology, Via Moruzzi 1, 56124 Pisa, Italy; aborghini@ifc.cnr.it (A.B.); pulignani@ifc.cnr.it (S.P.); mercuri@ifc.cnr.it (A.M.); turchi@ifc.cnr.it (S.T.); picano@ifc.cnr.it (E.P.)

**Keywords:** major adverse cardiac events, all-cause mortality, leucocyte telomere length, mtDNA^4977^ common deletion, coronary artery disease

## Abstract

Aging is one of the main risk factors for cardiovascular disease, resulting in a progressive organ and cell decline. This study evaluated a possible joint impact of two emerging hallmarks of aging, leucocyte telomere length (LTL) and common mitochondrial DNA deletion (mtDNA^4977^), on major adverse cardiovascular events (MACEs) and all-cause mortality in patients with coronary artery disease (CAD). We studied 770 patients (673 males, 64.8 ± 8.3 years) with known or suspected stable CAD. LTL and mtDNA^4977^ deletion were assessed in peripheral blood using qRT-PCR. During a median follow-up of 5.4 ± 1.2 years, MACEs were 140 while 86 deaths were recorded. After adjustments for confounding risk factors, short LTLs and high mtDNA^4977^ deletion levels acted independently as predictors of MACEs (HR: 2.2, 95% CI: 1.2–3.9, *p* = 0.01 and HR: 1.7, 95% CI: 1.1–2.9, *p* = 0.04; respectively) and all-cause mortality events (HR: 2.1, 95% CI: 1.1–4.6, *p* = 0.04 and HR: 2.3, 95% CI: 1.1–4.9, *p* = 0.02; respectively). Patients with both short LTLs and high mtDNA^4977^ deletion levels had an increased risk for MACEs (HR: 4.3; 95% CI: 1.9–9.6; *p* = 0.0006) and all-cause mortality (HR: 6.0; 95% CI: 2.0–18.4; *p* = 0.001). The addition of mtDNA^4977^ deletion to a clinical reference model was associated with a significant net reclassification improvement (NRI = 0.18, *p* = 0.01). Short LTL and high mtDNA^4977^ deletion showed independent and joint predictive value on adverse cardiovascular outcomes and all-cause mortality in patients with CAD. These findings strongly support the importance of evaluating biomarkers of physiological/biological age, which can predict disease risk and mortality more accurately than chronological age.

## 1. Introduction

Aging is one of the main risk factors for cardiovascular disease, resulting in a progressive organ and cell decline [1,2]. However, at an individual level, genetic and environmental factors may impact the biological aging process, resulting in a significant heterogeneity among subjects with the same age [3]. Consequently, chronological age alone may be a poor indicator of disease risk and mortality compared with biological age, which assesses the physiological state of an organism, resulting from the cumulative burden of endogenous and exogenous stressors, such as oxidative stress, inflammation, and lifestyle factors [3,4].

Biomarkers of aging may be a valuable tool to measure physiological age as well as providing additional prognostic/predictive information evaluating a biologic dimension presently ignored by current stratification risk [4,5,6]. Telomere shortening and mitochondrial dysfunction are two well-known hallmarks of aging and age-associated disease such as atherosclerosis [6].

Telomeres, specialized structures at the end of the eukaryotic chromosomes, protect the chromosome from deterioration or from fusion with neighboring chromosomes during cell replication [7]. After each cell division, the length of the telomere is shortened, and when a critical length is reached, the cell enters senescence or apoptosis. Additionally, a series of environmental stressors can accelerate telomere shortening [8,9]. 

In the last few years, numerous studies have shown a link between leucocyte telomere length (LTL) and cardiovascular disease [10,11,12].

Mitochondria are the major handlers for the cellular energy production. An adequate number of mitochondria per cell as well as a good mitochondrion functionality are required for the physiological homeostasis of cells and tissue. Mitochondrial damage, and in particular mitochondrial mutations such as the large 4977 kb mtDNA (mtDNA^4977^) deletion, occurs in many tissues during aging [13] and in a number of pathological conditions, including coronary artery disease (CAD) [14,15,16,17]. 

To date, telomere shortening and mitochondrial dysfunction have been examined mostly as independent contributors to CAD risk. However, there is growing evidence of a strong molecular linkage between telomere and mitochondrial dysfunction [18,19,20], supporting the hypothesis that both aging markers play a crucial role in the progression and evolution of vascular aging [21].

Accordingly, the purpose of this study was to evaluate the association between LTL and mtDNA^4977^ deletion, alone or in combination, with recurrent major adverse cardiovascular events (MACEs) and all-cause mortality in a relatively large population of patients with CAD.

## 2. Results

### 2.1. Baseline Characteristics and Correlations with LTL and mtDNA^4977^ Deletion

Demographic and clinical characteristics of the study population are shown in Table 1. 

The mean age of the participants was 64.8 ± 8.3 years and 87% were males. A total of 52% had a previous myocardial infarction (MI), and a previous revascularization was performed in 14% of the patients. The median value of LTL was a telomere repeat copy number/single copy gene copy number (T/S) ratio of 1.0 (T/S ratio: 0.69–1.40) and the median value of mtDNA^4977^ deletion was 0.56% (0.23%–1.0%). 

Baseline factors associated with a significantly shorter LTL were age (*p* = 0.04) and a previous history of MI (*p* = 0.02). The levels of mtDNA^4977^ deletion were also significantly higher in patients with a previous history of MI (*p* = 0.007). There was no association between levels of mtDNA^4977^ deletion and age or other patient characteristics. 

A significant inverse correlation, although weak, between LTL and mtDNA^4977^ deletion was observed (Sperman’s rho = −0.279, *p* < 0.0001; Figure S1 in Supplemental Materials). When the correlation between LTL and mtDNA^4977^ deletion in two different age groups (age of ≤65 and age of >65 years) was analyzed, the results remained significant across the subgroups (Figure S1 in Supplemental Materials). 

### 2.2. LTL, mtDNA^4977^ Deletion, and Outcome

During a mean follow-up of 5.4 ± 1.2 years, MACEs were 140 (18%), including 30 cardiac deaths, 36 nonfatal MIs, and 74 coronary revascularizations (coronary artery bypass graft (CABG) or percutaneous coronary intervention (PCI)). Eighty-six deaths (11%) were recorded. 

**Figure 1:**
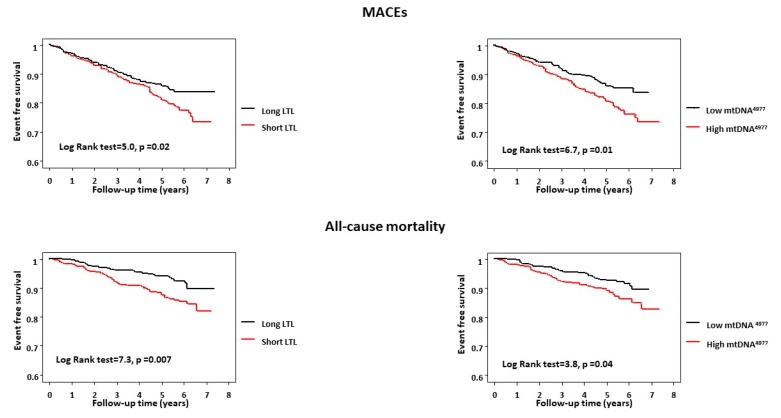
Kaplan–Maier plot of major adverse cardiovascular events (MACEs) and all-cause mortality for the leucocyte telomere length (LTL) and mtDNA^4977^ deletion. LTLs of <median value and ≥median value are stratified as short and long LTLs, respectively. mtDNA^4977^ deletion levels of >median value and ≤median value are classified as high and low mtDNA^4977^ deletion levels, respectively.

**Figure 2:**
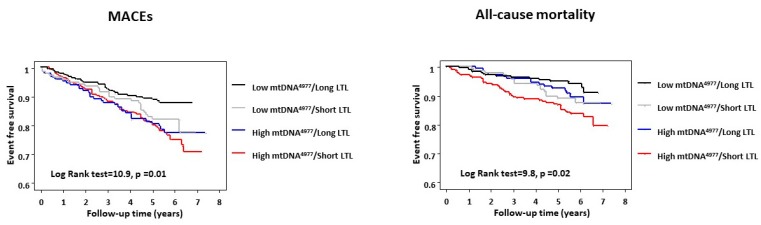
Kaplan–Meier plot of major adverse cardiovascular events (MACEs) and all-cause mortality for patients stratified in four groups according to LTLs and mtDNA^4977^ deletion levels. Short LTL = LTL of <median value; Long LTL = LTL of ≥median value; High mtDNA^4977^ deletion = mtDNA^4977^ deletion level of >median value; Low mtDNA^4977^ deletion = mtDNA^4977^ deletion levels of ≤median value.

Kaplan–Meier survival analysis showed that both shorter LTLs and higher mtDNA^4977^ deletion levels were independently associated with higher rates of adverse cardiovascular events (*p* = 0.02 and *p* = 0.01, respectively) and all-cause mortality events (*p* = 0.007 and *p* = 0.04, respectively) (Figure 1). There was a combined effect between the two biomarkers such that patients with both short LTLs and high mtDNA^4977^ deletion levels had the highest risk of adverse cardiovascular outcomes (log-rank test = 10.9, *p* = 0.01, Figure 2) and a substantial significant increase in all-cause mortality (log-rank test = 9.8, *p* = 0.02).

### 2.3. Regression Analyses and Combined Prognostic Value of LTL and mtDNA^4977^

In unadjusted Cox modeling, previous revascularization (HR: 1.6; 95% CI: 1.0–2.4; *p* = 0.04), short LTLs (HR: 1.5; 95% CI: 1.1–2.1; *p* = 0.02), and high mtDNA^4977^ deletion levels (HR: 1.6; 95% CI: 1.1–2.2; *p* = 0.01) were significantly associated with higher risk of MACEs.

Age (HR: 1.1; 95% CI: 1.0–1.2; *p* < 0.0001), hypertension (HR: 1.6; 95% CI: 1.1–2.5; *p* = 0.03), previous revascularization (HR: 1.9; 95% CI: 1.2–3.2; *p* = 0.01), multivessel disease (HR: 2.5; 95% CI: 1.6–4.0; *p* < 0.00001), creatinine levels (HR: 1.9; 95% CI: 1.1–3.3; *p* = 0.03), short LTLs (HR: 1.8; 95% CI: 1.2–2.8; *p* = 0.007), and high mtDNA^4977^ deletion levels (HR: 1.5; 95% CI: 1.1–2.4; *p* = 0.04) were also predictors of all-cause mortality.

After adjusting for age, gender, and other risk factors, patients with short LTLs had significantly more than 2-fold higher risk for the MACEs and all-cause mortality (Table 2). The associations for high mtDNA^4977^ deletion levels also remained significantly independent for both MACEs and all-cause mortality (Table 2). 

Table 3 shows multivariate Cox proportional hazard analyses for MACEs and all-cause mortality based on the combination of two aging biomarkers. After adjustment for the above-mentioned variables, patients in the short-LTL/high-mtDNA^4977^ group had significantly higher risk for MACEs (HR: 4.3; 95% CI: 1.9–9.6; *p* = 0.0006) and all-cause mortality (HR: 6.0; 95% CI: 2.0–18.4; *p* = 0.001) compared with the long-LTL/low-mtDNA^4977^ group. 

The global chi-square value of the clinical model for predicting MACEs was 13.3 (*p* = 0.1); after adding a short LTL, the global chi-square value increased to 18.3 (*p* = 0.03); mtDNA^4977^ deletion data also added significantly to the model (chi-square value = 24.0, *p* = 0.008). The global chi-square value of the clinical model for predicting all-cause mortality was 62.1 (*p* < 0.0001); the addition of short LTL increased the global chi-square value to 67.6 (*p* < 0.0001); the inclusion of mtDNA^4977^ deletion data also provided incremental information for predicting mortality (chi-square value = 70.7, *p* < 0.0001).

Furthermore, reclassification of patients, when predicting MACEs based on aging biomarkers with risk categories instead of clinical model alone, is summarized in Table 4. Net reclassification significantly (*p* = 0.01) improved for MACEs, when mtDNA^4977^ was incorporated into the risk model. The improvement in net reclassification improvement (NRI) was driven more by upward risk classification of patients with an MACE (38%) than by downward risk classification of patients without an MACE (27%).

Finally, sensitivity analyses showed that the results did not change if outliers of LTL and mtDNA^4977^ deletion were removed (data not shown). Moreover, when we further conducted stratified analyses based on age (age of <65 and age of ≥65 years), we observed that short LTLs and high mtDNA^4977^ deletion levels were associated with increased hazard for MACEs and all-cause mortality in both groups. However, shorter LTLs and higher mtDNA^4977^ deletion levels have shown a stronger association in younger patients (Table S1 in Supplemental Materials).

## 3. Discussion

To the best of our knowledge, this is the first study to investigate the prognostic value of telomere length and mtDNA^4977^ deletion levels in a relatively large cohort of patients with CAD. In this study, LTL was significantly inversely correlated with mtDNA damage, supporting the hypothesis that these two aging biomarkers can act independently of one another. Moreover, our findings clearly showed that both markers are independent predictors of MACEs and all-cause mortality. Additionally, patients carrying both short LTLs and high mtDNA^4977^ deletion levels had the highest risk of adverse outcomes, highlighting the importance of an integrated assessment of nuclear and mitochondrial genomic functions as an individual clinical index of cellular decline.

### 3.1. Comparison with Other Studies

During the last few years, numerous clinical studies have investigated the relation between LTL and the risk of CAD and ischemic events, as recently analyzed in two independent meta-analyses [22,23]. Indeed, Haycock et al. conducted a meta-analysis of 24 studies involving 43,725 participants and 8400 patients with cardiovascular disease reporting an inverse association between LTL and nonfatal MI and CAD death, independently of conventional vascular risk factors [22]. The association was consistent after stratification for relevant subgroups, such as different mean ages and sex distributions, and across prospective and retrospective studies. In the meta-analysis of 27 studies by D’Mello and colleagues, a significant association was found between shortened LTL and MI and stroke, suggesting that LTL attrition is a potential marker of plaque rupture and acute ischemic events [23].

Furthermore, a genome-wide meta-analysis revealed an association between single-nucleotide polymorphisms associated with short LTLs and an increased risk of CAD, supporting a causal role of telomere erosion in the pathogenesis of diseases [24].

Additionally, previous studies showed a significant association between LTLs and adverse outcomes in patients with CAD [25,26], especially for younger patients [27].

The role of mitochondrial damage in the pathophysiology of atherosclerosis is still being extensively discussed [28,29], but several recent studies have shown a critical role for mitochondrial reactive oxygen species (ROS) and mtDNA damage in animal models of atherosclerosis [30,31].

Moreover, human studies have demonstrated a correlation between mtDNA damage and the development and the progression of atherosclerosis [32,33]. Specifically, mtDNA^4977^ deletion accumulated in atherosclerotic vascular walls and diseased cardiac tissue [15,34]. 

Our previous study showed that the levels of blood mtDNA^4977^ deletion were higher in CAD patients than in healthy age-matched subjects [16]. More recently, we showed an independent prognostic value of high levels of mtDNA^4977^ deletion on MACEs and all-cause mortality, highlighting the importance of mitochondrial quality rather than mitochondrial quantity in the cardiovascular field [17]. 

In line with all these observations, the present study confirms a major independent role of both telomere length and mtDNA integrity in predicting adverse outcomes after an event of myocardial ischemia. 

Importantly, a complicated “telomere-mitochondria interplay” has been recently hypothesized, where the dysfunction of one worsens the condition of the other amplifying and accelerating cell health decline, supporting a unifying mechanism for cellular aging [18,19,20].

It is known that mitochondrial dysfunction as well as increased mitochondrial density and biogenesis leads to an abnormal generation of reactive ROS, strictly associated with chromosomal instability through telomere attrition [35]. On the opposite side, mitochondria can become dysfunctional when telomeres are shortened as a consequence of a p53-dependent repression of the peroxisome proliferator-activated receptor gamma coactivator 1α (PGC-1α), a master regulator of mitochondrial biogenesis and function [18,36]. 

The findings of our study are only partly consistent with this pathogenic theory, showing a slight relationship between telomere shortening and high mtDNA^4977^ deletion levels in peripheral blood of CAD patients and their independent effects on the long-term outcome. In light of this, other explanations need to be explored. 

Importantly, recent studies ascribe a fundamental double role for telomerase, classically known as an enzyme that maintains a telomere length in nuclear DNA, which can drive both mtDNA damage and telomere shortening. Indeed, TERT, the catalytic subunit of telomerase, can reversibly translocate from the nucleus to the mitochondria in response to stressors. In mitochondria, TERT may regulate the levels of ROS reducing mtDNA damage. Nevertheless, a protective and pathological role of telomerase may be a result of cellular and tissue specificity. Indeed, telomerase activation reduced ROS levels (and thus inflammation) in the endothelium, whereas increased telomerase activity within the vascular smooth muscle layer resulted in abnormal proliferation and vascular remodeling [37]. Additionally, in this study, we analyzed these two aging biomarkers in peripheral leukocytes that represent a heterogeneous population of cells, including monocytes, granulocytes, and lymphocytes. Therefore, we cannot exclude that a stronger link between telomere length and mtDNA damage may exist in specific vascular cell populations, more strictly driving CAD development and progression.

### 3.2. Study Limitations

Some limitations in our study have to be taken into account. First, as this observational study, we cannot exclude the possibility of uncontrolled confounding factors or selection biases. Second, our cohort is from a single institution and not having external validity. Third, most of patients have existing CAD or previous revascularization/MI, and we did not investigate temporal changes in cellular aging markers that may occur in response to medical therapy or disease evolution, affecting the risk prediction.

Despite its limits, this study strongly suggests the importance of evaluating biomarkers of physiological/biological age in order to identify patients at increased risk for recurrent cardiovascular events, who are currently not adequately protected with conventionally available risk factors.

## 4. Materials and Methods

### 4.1. Study Population and Follow-Up

We studied 770 Caucasian patients (673 males) with known or suspected CAD, who were enrolled in a large cohort of Genetic Mapping for Assessment of Cardiovascular Risk (GENOCOR). The study design with inclusion criteria have been described elsewhere [17,38].

Briefly, data were collected on age, gender, diabetes (fasting plasma glucose: >120 mg/dL), hypercholesterolemia (plasma cholesterol: >220 mg/dL), obesity (body mass index: >30 kg/m^2^), arterial hypertension (systolic blood pressure: >140 mmHg and/or diastolic pressure: >90 mmHg). Smokers were classified as individuals who smoked at least 3 cigarettes per day at the time of analysis, past smokers had quit smoking for at least 6 months, and nonsmokers were individuals who had never smoked. Smoking patients were the combined groups of past and current smokers. Data on left ventricular function (LVEF) were obtained by echocardiography or left ventricular angiography. All patients were subjected to a follow-up program to certify MACEs defined as coronary-related death, nonfatal MI, and coronary revascularization (i.e. CABG and PCI). The cause of death was derived from medical records or death certificates provided by local health authorities. The definition of cardiac death required the documentation of either significant arrhythmias, cardiac arrest, or death attributable to congestive heart failure or MI in the absence of any other precipitating factor. All patients were censored after the first adverse cardiovascular event during the follow-up. The study was approved by the local ethics committee (Comitato Etico Sperimentazione Farmaco - Azienda Ospedaliera Universitaria Pisana, Italy). ClinicalTrials.gov Identifier is NCT01506999 (January 10, 2012). Written informed consent was obtained from all patients.

### 4.2. Leukocyte Telomere Length and mtDNA^4977^ Deletion Measurement

Total DNA was extracted from peripheral blood leukocytes by using the QIAGEN BioRobot® EZ1 System. Both the LTL and mtDNA^4977^ deletion levels were measured by using quantitative real-time methods (CFX384 Touch Real-time PCR detection system, Bio-Rad, Hercules, CA, USA) following previously described protocols [17,39]. Briefly, LTL was measured in genomic DNA by determining the T/S ratio). A relative telomere length was calculated by the equation: T/S ratio = 2^–ΔCt^, where Ct is a threshold cycle and ΔCt = Ct × telomere − Ct × single copy gene. The T/S ratio reflected the average length of the telomeres across all leukocytes. Similarly, the levels of mtDNA^4977^ deletion (nucleotides between 8.470 and 13.447 bp) was determined through the amplification of the *NDI1* gene in an undeleted region of mtDNA (mtNDI1) and the remaining fragment after mtDNA^4977^ deletion. The difference in the average threshold cycle (Ct) number values was used for the measurement of relative content. The percentage of the mtDNA^4977^ deletion was calculated as: 2^–ΔCt^ × 100%, where ΔCT = Ct × mtDNA^4977^ − Ct × mtNDI1. 

For both LTL and mtDNA^4977^ deletion assay, a standard curve was included in each plate using pooled human DNA from 10 healthy donors in order to assess qRT-PCR efficiency. All samples were run in triplicates to evaluate the intra-assay precision. 

### 4.3. Statistical Analyses

Categorical data, expressed as frequencies and percentages, were compared using the Fisher’s exact test. Normally distributed continuous variables are presented as mean ± standard deviation (SD) and as the median (25th–75th percentile) in a non-normal distribution. Continuous variables were compared using Student’s t test and Mann–Whitney U test for data with a normal distribution and a non-normal distribution, respectively. Categorical variables were compared using the chi-squared test. The Spearman’s rank correlation was used to test the association between aging markers and other continuous parameters. Kaplan–Meier survival analysis was performed to compare the difference in survival rate between patients with short and long LTLs (<median value and ≥median value, respectively), and high and low mtDNA^4977^ deletion levels (>median value and ≤median value, respectively) using the log rank test. Cox proportional hazard models were used to assess the predictive value of each variable. All multivariate models included age, gender, smoking, hypertension, hypercholesterolemia, diabetes, obesity, multivessel disease, previous MI, previous CABG, previous PCI, and LVEF. 

To assess the added prognostic value of LTL and mtDNA^4977^ deletion, the clinical model was compared with a model, in which aging biomarkers were not included. The global chi-square statistic was calculated for both models and compared using the likelihood-ratio test. Additionally, the increased discriminative value of aging biomarkers was also estimated by the NRI approach [40], applying pre-specified tertile categories of risk: low (<8%), intermediate (8% to 12%), and high (>12%). A *p*-value of <0.05 was considered statistically significant in this study.

## 5. Conclusions

In conclusion, LTL and mtDNA^4977^ deletion showed an independent and joint predictive value on adverse cardiovascular outcomes and all-cause mortality in patients with CAD.

## Figures and Tables

**Table 1:** Demographic and clinical characteristics of the study population.

Characteristic	Value
Mean age (years ± SD)	64.8 ± 8.3
Male, *n* (%)	673 (87)
Current smoker, *n* (%)	477 (62)
Obesity, *n* (%)	225 (29)
Hypercholesterolemia, *n* (%)	533 (69)
Hypertension, *n* (%)	370 (48)
Diabetes, *n* (%)	112 (15)
LVEF (%)	50 (48–58)
*Number* of diseased coronaries	
1, *n* (%)	398 (52)
2, *n* (%)	239 (31)
3, *n* (%)	133 (17)
Previous revascularization, *n* (%)	111 (14)
Previous MI, *n* (%)	398 (52)
Creatinine (mg/dL), described as (mean ± SD)	1.1 ± 0.3
Medical treatment	
Aspirin, *n* (%)	708 (92)
Statin, *n* (%)	554 (72)
ACEI/ARB, *n* (%)	209 (27)
Beta-blocker, *n* (%)	339 (44)
Calcium channel blocker, *n* (%)	285 (37)
LTL, described as a T/S ratio	1.0 (0.69–1.40)
mtDNA^4977^ deletion (%)	0.56 (0.23–1.0)

Values are mean ± SD, *n* (%), or median (25th–75th percentile range). LTL: leucocyte telomere length; T/S ratio: ratio of telomere repeat (T) copy number to single copy gene (S) copy number; LVEF: left ventricular ejection fraction; MI: myocardial infarction; ACEI: angiotensin-converting enzyme inhibitor; ARB: angiotensin receptor blocker.

**Table 2:** Multivariate Cox regression analysis of the association of LTL and mtDNA^4977^ with major adverse cardiovascular events (MACEs) and all-cause mortality.

	MACEs		All-Cause Mortality	
	HR (95% CI)	*p*-Value	HR (95% CI)	*p*-Value
Age, 1-year increase	1.0 (0.9–1.0)	0.99	1.1 (1.1–1.2)	<0.0001
Male	0.9 (0.4–2.1)	0.80	0.9 (0.3–2.9)	0.80
Smoking	1.2 (0.7–2.1)	0.56	2.6 (1.2–6.0)	0.02
Obesity	1.3 (0.7–2.2)	0.44	1.1 (0.5–2.3)	0.71
Hypercholesterolemia	0.7 (0.4–1.3)	0.33	0.7 (0.4–1.4)	0.32
Hypertension	1.1 (0.6–1.8)	0.85	1.2 (0.6–2.3)	0.57
Diabetes	1.3 (0.7–2.6)	0.40	1.4 (0.6–3.1)	0.41
Ejection fraction (%)	1.0 (0.9–1.0)	0.49	0.9 (0.9–1.0)	0.09
Multivessel disease	1.8 (0.8–2.4)	0.18	1.2 (0.7–4.5)	0.54
Previous revascularization	1.6 (0.8–3.1)	0.18	2.0 (0.8–4.8)	0.11
Previous MI	0.8 (0.4–1.3)	0.32	0.7 (0.3–1.4)	0.32
Creatinine	1.3 (0.6–2.8)	0.56	1.8 (0.7–4.6)	0.22
Short LTL	2.2 (1.2–3.9)	0.01	2.1 (1.1–4.6)	0.04
High mtDNA^4977^	1.7 (1.1–2.9)	0.04	2.3 (1.1–4.9)	0.02

**Table 3:** Multivariate Cox proportional hazard model for risk of major adverse cardiovascular events (MACEs) and all-cause mortality in groups based on short LTLs and high mtDNA^4977^ deletion.

	MACEs			All-Cause Mortality		
	HR	95% CI	*p*-Value	HR	95% CI	*p*-Value
long LTL/low mtDNA^4977^ (*n* = 126)	1.00		—	1.00		—
long LTL/high mtDNA^4977^ (*n* = 76)	2.3	0.9–6.2	0.09	3.4	0.9–12.6	0.07
short LTL/low mtDNA^4977^ (*n* = 84)	2.7	1.1–7.5	0.04	2.8	0.7–10.4	0.12
short LTL/high mtDNA^4977^ (*n* = 134)	4.3	1.9–9.6	0.0006	6.0	2.0–18.4	0.001

Adjusted for age, gender, smoking, hypertension, hypercholesterolemia, diabetes, obesity, multivessel disease, previous MI, previous coronary artery bypass graft, previous percutaneous coronary intervention, creatinine levels, and left ventricular ejection fraction.

**Table 4:** Net reclassification improvement (NRI) for prediction of major adverse cardiovascular events by addition of the mtDNA^4977^ and LTL to a baseline clinical model.

	Direction of Reclassification			
	Upward	Downward	NRI (SE)	*p*-Value
mtDNA^4977^				
*Event*	53 (38)	185 (27)	0.18 (0.07)	0.01
*Non-event*	28 (20)	185 (27)		
LTL				
*Event*	51 (36)	196 (31)	0.09 (0.07)	0.2
*Non-event*	37 (26)	189 (30)		
mtDNA^4977^ + LTL				
*Event*	57 (41)	203 (32)	0.19 (0.07)	0.01
*Non-event*	33 (24)	211 (33)		

SE: standard error.

## Supplementary Materials

Supplementary materials can be found at https://www.mdpi.com/1422-0067/20/21/5508/s1.
