# Supplementary material for: Independent and Combined Effects of Telomere Shortening and mtDNA4977 Deletion on Long-term Outcomes of Patients with Coronary Artery Disease
Source: Int J Mol Sci. 2019 Nov 5;20(21):5508. doi: 10.3390/ijms20215508 (PMC6862167; doi:10.3390/ijms20215508)
Supplement: Supplementary file 1 [file ijms-20-05508-s001.pdf]

Figure S1. Scatter plots showing the association of leukocyte telomere length (LTL) with mtDNA4977 deletion levels (as continuous variables) in the whole population and in the population stratified by the mean of age (65 years).

Whole population (n=770)

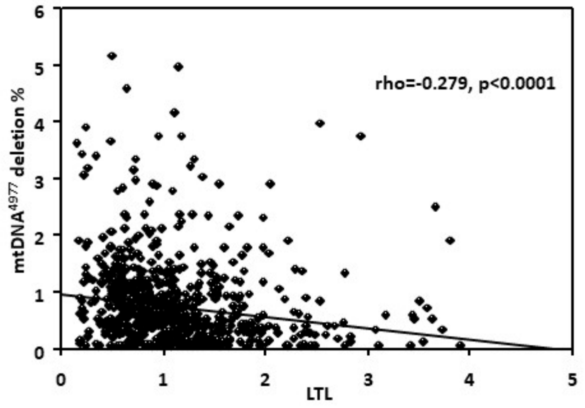

Age ≤ 65 years (n=381)

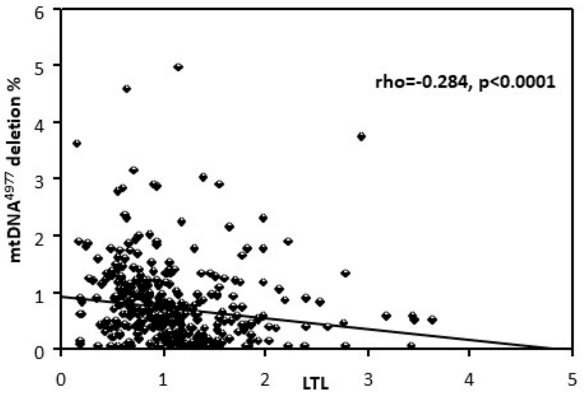

Age > 65 years (n=389)

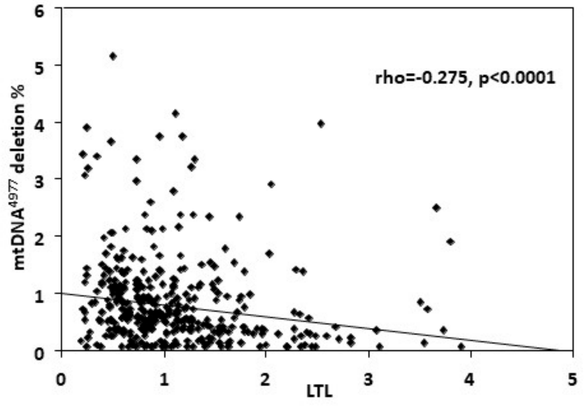

Table S1. Cox regression analysis of the association of LTL and mtDNA^{4977} with major adverse cardiovascular events and all-cause mortality in stratification analysis by age.

	MACEs		All-cause mortality	
	**HR(95% CI)**	**p-value**	**HR(95% CI)**	**p-value**
***Age < 65 years* (n=381)**				
long LTL / low mtDNA^{4977}	Reference	—	Reference	—
long LTL / high mtDNA^{4977}	1.8(0.8-3.7)	0.11	3.7(0.3-41.4)	0.3
short LTL / low mtDNA^{4977}	1.3(0.6-3.2)	0.5	13.96(1.5-130.2)	0.02
short LTL / high mtDNA^{4977}	2.4(1.3-4.7)	0.007	17.7(2.2-142.4)	0.006
***Age ≥ 65 years* (n=389)**				
long LTL / low mtDNA^{4977}	Reference	—	Reference	—
long LTL / high mtDNA^{4977}	2.0(1.1-4.3)	0.03	1.5(0.7-3.3)	0.16
short LTL / low mtDNA^{4977}	1.6(0.8-3.3)	0.2	0.9 (0.5-2.1)	0.70
short LTL / high mtDNA^{4977}	2.0(1.1-3.9)	0.03	1.7(0.9-3.2)	0.06

Multivariable model adjusted for confounders.
